# Impact of Educational Technology on Teacher Stress and Anxiety: A Literature Review

**DOI:** 10.3390/ijerph18020548

**Published:** 2021-01-11

**Authors:** José-María Fernández-Batanero, Pedro Román-Graván, Miguel-María Reyes-Rebollo, Marta Montenegro-Rueda

**Affiliations:** Department of Teaching and Educational Organization, University of Seville, 41013 Seville, Spain; proman@us.es (P.R.-G.); mmreyes@us.es (M.-M.R.-R.); mmontenegro1@us.es (M.M.-R.)

**Keywords:** educational technology, teacher burnout, anxiety disorders, mental health, review

## Abstract

Educational technology has become an increasingly important element for improving the teaching and learning process of students. To achieve these goals, it is essential that teachers have the skills they need to be able to introduce technology into their teaching practice. However, this is often overwhelming and stressful for many of them. The aim of this review was to find out how research on teacher stress and anxiety associated with the use of educational technology was proceeding. A systematic review was conducted using the Preferred Reporting Items for Systematic Reviews and Meta-Analyses (PRISMA) guidelines through the following bibliographic databases: PubMed, Web of Science, and Scopus. Sixteen articles were found from the review. The main findings show that teachers present high levels of anxiety or stress due to their use of educational technology in the classroom. Among the conclusions, the need for research on different strategies to prevent the emergence of these anxiety and stress symptoms in teachers stands out.

## 1. Introduction

Studies on technologies in education have focused mainly on improving the learning processes of students. However, research on how teachers have been affected by the emergence of the technologies that make improved student learning possible is scarce. Technologies have transformed the manner in which individuals work, since technologies are support tools—improving individuals’ working and personal activities and transforming them into more efficient people. Furthermore, the use of technology allows the freeing up of time for individuals such as teachers to carry out other activities, independent of their professions. However, technologies are also responsible for alterations in people’s lives, which are not always suitable since they disrupt personal and interpersonal relationships and even affect health. The incorporation of technology may become a focus of tension and anxiety among teachers, influencing their daily lives. Often, the inclusion of educational technology is demanded despite the lack of technical resources and equipment necessary for its correct didactic use. These situations culminate in conflicts between teachers, as well as in their relationships with colleagues or with other people involved in the environment, eventually producing, in the worst case, damaged personal and interpersonal relationships that affect their health.

Currently, there is interest in incorporating technology in the classroom due to the multiple benefits it can bring to students. However, the reality shows that its use may also be negative for teachers because it could imply changes in their teaching methods or pressure to acquire technological skills, leaving sequelae such as physical, social, and psychological problems [1].

Among the main symptoms that teachers present due to the pressure to which they are exposed related to the use of educational technology are stress and anxiety [2]. Considering all of the different conceptualizations, stress in the workplace refers to the response adopted by individuals when faced with a threatening situation in the workplace [3], resulting from different factors that are aggravated by the use of new technologies [4]. Increased demands on the use of technology can also develop other emotions, such as anxiety. Although there is no single definition of this term, it can be associated with other terms such as fear or distress [5]. Anxiety usually arises in situations of ignorance. Specifically, in the field of educational technology, some studies found that students do not show technostress, a type of stress related to the implementation of technologies, due to students’ extensive knowledge of technologies [6]. However, for teachers this is not usually the case. Incorporating technology into their teaching practices without being aware of the didactic possibilities that technology offers, a lack of training in educational technology, or resistance to its use produces fatigue in the professional and working environments [7]. The appearance of these symptoms can be associated with the improper use of technologies, as well as the avoidance of their use [8]. Among the symptoms, we can also find “burnout syndrome”, which is related to exhaustion and burnout due to increasing demands [9]. In the pedagogical context, burnout syndrome in teachers can affect their level of commitment at work.

Considering the above, it is important to investigate the overall state of research in the field of teacher stress and anxiety associated with the use of educational technology through a review in different databases (PubMed, Web of Science, and Scopus), synthesizing the main trends and emerging areas of research to understand how teachers properly manage stress and anxiety when using educational technology.

This paper is justified by pointing out that the technological changes faced by teachers are generating new working conditions, which imply greater stress for teachers, affecting their living standards, their families, their interpersonal relationships at their workplace, and society as a whole. Moreover, research on technology in education has focused mainly on improving students’ learning processes. However, research on how teachers have been affected by the emergence of educational technology in their daily teaching work is scarce and current statistical data are hardly available. Therefore, this study aimed to relate the influence that educational technology has played on teachers’ mental health, specifically on their levels of stress and anxiety. 

This paper is organized in three parts. First, we present a theoretical point of view based on a review of available literature and research in this field. Second, we present the findings and results of studies aimed at examining the link between teachers’ mental health and the use of technologies in the classroom. Third, the paper concludes with recommendations and future areas of research that address the gaps found in the current literature.

One of the limitations that we found in the review of the literature evaluating the impact of educational technologies on stress and anxiety in teachers was that the use of technologies has been changing in recent years, with each change being more ubiquitous and familiar.

Consequently, this study aimed to research a field little explored so far but which is becoming increasingly important. Through the systematic review, the countries with the greatest scientific output in the field were analyzed, as well as the type of methodology used, all with the aim of exploring the main factors related to stress and anxiety in teachers due to the integration of educational technologies, as well as an aim of detecting and identifying future areas of research in this field.

## 2. Methods 

The main objective of the study was to explore the evidence in publications reporting on stress and anxiety in teachers provoked using educational technology.

### 2.1. Search Strategy

A systematic search of the literature was carried out using the following databases: PubMed, Web of Science (hereinafter WoS), and Scopus. We chose to limit the review to the most recent studies, including only articles published in the last 15 years (January 2005–December 2019) and applying the selection of the following search terms and key words extracted from the thesaurus of ERIC (Education Resources Information Center) descriptors: stress, anxiety, educational, information and communication technologies, technostress, and burnout. To provide rigor within the research, the keywords were crossed with the Boolean operator OR/AND. 

The systematic review was conducted considering PRISMA (Preferred Reporting Items for Systematic Reviews and Meta-Analyses) statement guidelines [10]. Based on the characteristics of this study, descriptive and qualitative techniques were used, as well as semantic applications to the analysis of social networks [11] through visual representation with VOSviewer software (Centre for Science and Technology Studies (CWTS), Leiden, The Netherlands). 

### 2.2. Inclusion Criteria and Selection of Studies

Studies were selected in the review if the following inclusion criteria were met: (a) written in English or Spanish, (b) addressed the issue of stress, burnout, or mental health in teachers using educational technology, (c) appeared in peer-reviewed journals, and (d) were published openly. Studies were excluded if they: (a) were not available in their entirety, (b) were not related to the field of education, (c) were conference proceedings, reviews, book chapters, books, or other types of publications, and (d) were duplicated articles in different databases. 

**Figure 1:**
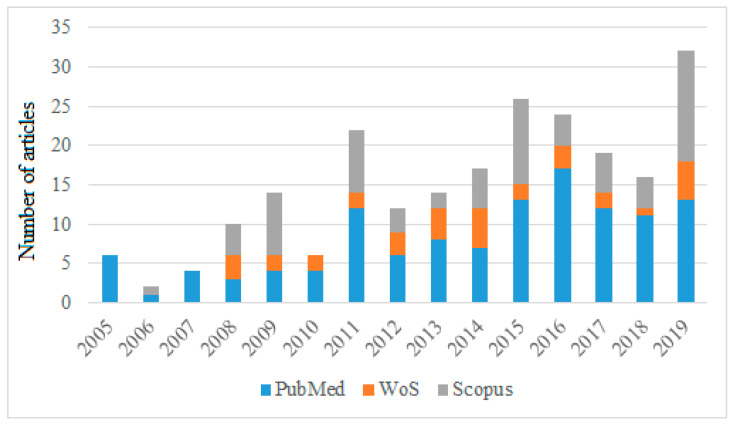
Distribution and progress of papers per year in different databases. WoS: Web of Science.

During a first search, considering the selected inclusion criteria, a total of 223 records were retrieved from all the selected databases (121 publications in PubMed, 33 in Web of Science, and 69 in Scopus). Additionally, the reference lists of the selected articles were reviewed. Figure 1 shows the distribution and evolution of the number of articles published in the 2005–2019 period in the databases analyzed.

The figure (Figure 1) shows the growth in published papers involving educational technology and teacher stress and anxiety over the last 15 years. We can see how publications that included our search criteria became more and more of interest from 2015, increasing their production. 

**Figure 2:**
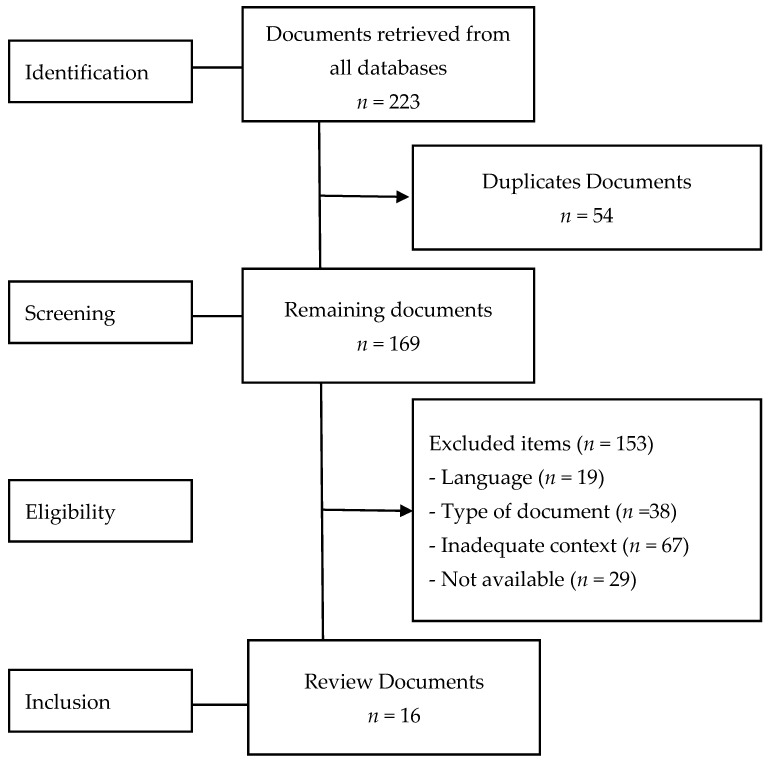
Sample selection flowchart.

After an initial screening, a total of 54 repeated records were excluded. Once the remaining records were examined, 153 documents that did not meet the inclusion criteria were eliminated because 19 were published in a language other than English or Spanish, 38 were an excluded type of document (doctoral theses, books, communications, conference proceedings, and technical reports), 67 belonged to areas other than education, and 29 were not available in full text, leaving 16 records finally included in the review. Figure 2 shows a flow chart of the study selection process.

### 2.3. Data Extraction

Subsequently, the selected articles were coded for the analysis and discussion procedure using a database from which information was interpolated into graphs and tables. Table 1 shows a summary of the detailed analysis of the 16 articles selected for review, indicating all the information relevant for the review contained in the inclusion criteria.

## 3. Results

The results of this study are presented in two phases. First, we describe the results achieved from the analysis of the documents; then, the results are presented using a visual representation of the analysis of keyword graphs extracted from different databases to be able to extract the main trends when studying teachers’ stress and anxiety related to the use of educational technology.

There were only 16 studies published between 2005 and 2019 included in this review study. Examining the relevant literary production over the last 15 years (Table 1), it can be seen that the majority (80% of the publications) were published from 2015 onward. This suggests that this issue is currently receiving increased attention, suggesting an emerging area of research.

**Figure 3:**
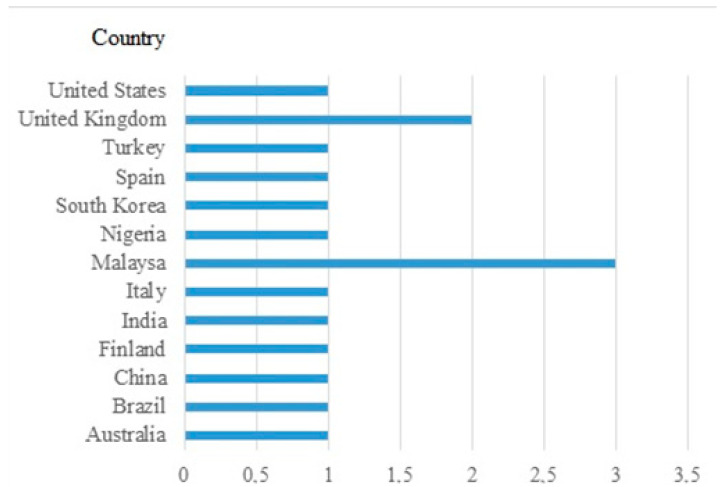
Distribution of publications by country.

In an analysis by country (Figure 3), we can detect that the greatest scientific production in this field has been developed in Malaysia (*n* = 3), followed by the United Kingdom (*n* = 2).

The methodology used in the papers analyzed provides an overview of how research and reflection on teacher stress and anxiety related to the use of educational technology is being addressed. Most publications (14 publications) had a quantitative approach, as we can see in Table 1. Articles with a mixed or qualitative methodology were fewer.

The selected studies explored the negative effects of the use of educational technology on teachers’ mental health. Considering the different studies, the results point mainly to symptoms of exhaustion, increased anxiety levels, or perceived stress when using these technological tools was caused by a lack of teacher training. To a lesser extent, there are also studies that reported teacher fatigue from having to use educational technology in their teaching practices. 

### 3.1. Description of the Studies Examined

The studies included in this systematic review shared the objective of linking the use of educational technology and teacher professionalism. More specifically, through different approaches they examined the impact of using educational technology on teachers in both their personal and professional lives. Many studies found that these new challenges that teachers must face affect their social and personal well-being. For example, the study of Pillay, Goddard and Wills [12] analyzed the relationship between burnout and competence for a sample of mid-career teachers in primary and secondary schools in Queensland. Their findings suggest that teacher burnout and anxiety arise when they are not comfortable in the workplace, since they do not feel “competent”. In addition, they found that many teachers felt that their efforts in developing their teaching practices were outweighed by their own rewards. Also related to teachers’ burnout when they feel overwhelmed by the demands and functions related to their teaching role is the study conducted by Tucker [22], called “To Stem Teacher Burnout, Go Digital”. The author believes that the promotion of the student engagement in technology has an impact on both teacher performance and training, causing them anxiety and stress. The large number of demands and obligations linked to their professional role forces teachers to work after school hours and in their personal lives to respond to them. An example found in this study was that teachers tended to “feel pressure to do it all” (p. 1). This paper represents a changing mindset about the teaching profession, turning the transmitting role into a guiding role for both teaching and learning processes. There, technology becomes an ally to monitor students’ autonomous performance, and the “workloads” between students and teachers are levelled out, decreasing teachers’ anxiety.

However, many of the papers included in the review arose from the pressure that teachers feel to use educational technology but do not feel prepared to do so. For example, Al-Fudail and Mellar [13] conducted their research on the ‘teacher–technology environment interaction model’ to examine whether teachers were experiencing technology stress when they had to incorporate educational technology into their teaching practices. Their results confirmed that teachers suffer from technology stress when using educational technology and indicate that the main factors are the effort to explain how technology works to students, the training required to remodel teaching practices, the problems with the operation of school software and facilities, and the lack of support for implementing technology in the classroom. In contrast, research by La Paglia et al. [14] focused exclusively on the relationship between technological self-efficacy and stress, stating that this stress was mainly caused by a lack of technological knowledge and a fear of the new. Similarly, Awofala et al. [23] analyzed teachers’ technological stress based on three variables: the degree of technology integration into teaching and learning are attitudes toward computers, computer anxiety, and computer self-efficacy. Their results show significant correlations between attitudes toward educational technology, computer anxiety, and computer self-efficacy. Related to this issue is the research of Agbatogun [15], who analyzed the relationship between anxiety and self-concept with the use of educational technology from a gender perspective. In their findings they found no significant differences in the emergence of technostress according to gender, coinciding with similar research by Çoklar et al. [18] in Turkey with teachers of different educational stages.

The introduction of educational technology to innovate in the classroom is a great challenge for teachers who do not feel “competent”. In turn, the attitude of teachers to use educational technology can be predicted by the union of self-concept, anxiety, and gender factors. In this regard, the keys to “compensate” and solve these problems are located in technological skills training.

By contrast, Bolandifar & Noordin [16] did find significant differences with respect to technostress according to gender. Their study—developed with a sample of 160 in-service teachers in Iran—showed that the attitude of teachers toward the use of educational technology influenced the prevalence of having or not having stress. For them, the best way to reduce anxiety and stress generated by technology is to try to change teachers’ attitudes to using it. Supporting technology’s use in the classroom, giving teachers the tools they need, and making them see the possibilities technology offers is the best way to reduce their anxiety. Similarly, the research by Revilla-Muñoz et al. [21] carried out in Spain places technology training as the best option for reducing technostress in teachers.

In India was another of the studies included in this systematic review. Specifically, the research from Jena [17] analyzed the relationship between technostress creators, technostress inhibitors, and technostress effects among Indian academicians in higher education. Their results show that technostress creators appear because of insecurity and lack of knowledge of teachers (individual); technostress inhibitors—related to the identification, involvement, and loyalty with the school organization—decrease the effects of the appearance of technostress. Furthermore, the author provided some suggestions to combat the emergence of technostress (p. 1022): (a) keep a positive attitude toward innovation, (b) avoid resistance to technological change due to its emotional connection, being more likely to the emergence of stress, (c) know and use stress reduction techniques, (d) upper management should provide the necessary training and support for teachers to introduce educational technology into their practices, and (e) infrastructure is needed to incorporate educational technology into the teaching and learning processes. This last issue is one for which an administration must undertake significant responsibility to ensure the effectiveness of instructional processes with the use of educational technology. Additionally, in the field of higher education, a study of Wang and Li [27] found that university management regarding the use of educational technology influenced the technostress suffered by teachers. 

The study by Goebel and Carlotto [24] went one step further by pointing out that the technostress of teachers arises from their feelings of lack of autonomy and lack of training and teachers’ need for pedagogical renewal and updating, communication changes with students, and work–family balance.

Along this line, the study by Syvänen et al. [20] found a direct relationship between the use of educational technology and the generation of techno-strengths, identifying that teachers prefer not to incorporate educational technology into instructional processes because it causes them anxiety and stress when they have to do so. Specifically, the authors examined the emergence of technostress according to sociodemographic criteria and the attitude of teachers in the integration of educational technology. Their results showed that the key predictors of technostress were Information and Communication Technology (ICT) competence, the concordance of the educational use of educational technology with the teaching style, school support, and attitudes to the educational use of educational technology. Conversely, teachers who had a positive attitude toward educational technology, whose teaching was technology-friendly, and who enjoyed school support showed lower levels of technostress.

Unlike previous studies, the study by Othman & Sivasubramaniam [26] was more general and discussed the levels of anxiety, stress, and depression associated with the teaching profession. Their manuscript points out that the general teaching challenges and their additional new demands—especially those related to technology—make the levels of stress and anxiety grow exponentially.

In Korea, Joo et al. [19] conducted a similar study with high school teachers. Its purpose was to analyze the attitude of teachers to incorporate technology, the infrastructure of schools to introduce educational technology, and the technostress of teachers. The study was conducted from the perspective of the Technological Pedagogical Content Knowledge (TPACK) model using four validated questionnaires. In their findings they found that the TPACK model and the support of the school influenced the existence of technostress in teachers and their disposition toward educational technology.

Finally, the study by Hassan et al. [25] linked the emergence of technostress in teachers with digital literacy. Simultaneously, they state that the exponential increase of teachers’ functions affects the appearance of stress and anxiety in them, especially in older people. Commitment to the teaching profession and the educational institution, as well as training in educational technology, stand out as potential factors for reducing stress.

### 3.2. Analysis of the Papers Included Using VOSviewer

**Figure 4:**
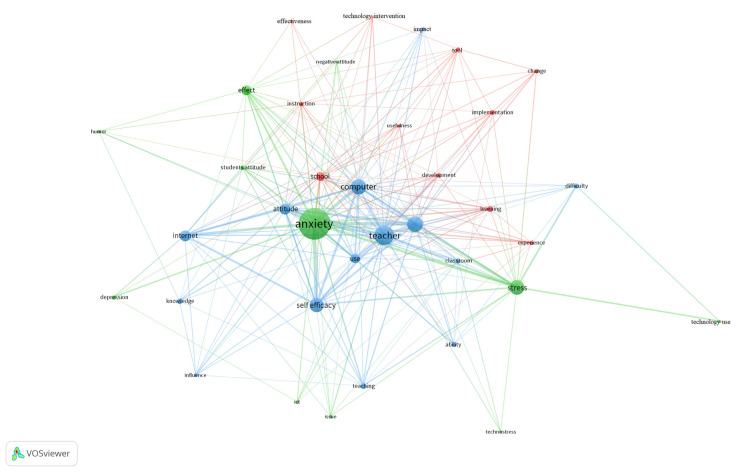
Bibliometric map of the 36 KeyWords Plus (KW+) representation.

Regarding the stressors or factors associated with teachers’ stress and anxiety due to the use of educational technology that appear in the literature, as we can see in Figure 4, the factors are mainly focused on the lack of training and education in technology (50%), the need for constant updating and innovation (25%), pressure to be constantly using the technologies (18.75%), and insecurity when using educational technology (6.25%).

Second, once the descriptive and quantitative analysis of the selected documents were carried out, the results of the analysis of the relationships established between the key words extracted automatically or by KeyWords Plus (KW+) from the different databases were presented using the VOSviewer program.

Thus, the 16 studies collected in the period 2005–2019 were analyzed, from which a total of KW+ was obtained. Figure 4 clearly shows the three groups or clusters that were generated according to the degree of similarity of the KW+. The size of each circle or node represents the importance that each keyword holds in this review, while the links or distance reflect the relationships between two nodes. Here, a total of 36 KW+ was obtained.

The three thematic clusters that defined the main research themes in this field are: 

Cluster1: Identified in green, this cluster is related to the effects that the incorporation of educational technology in education has had on teachers. This approach focuses on the main part of this study. It can be observed that in this cluster appear terms like anxiety, stress, depression, and technostress.

Cluster2: Appearing in blue, this cluster is related to the factors or variables that increase the levels of stress and anxiety of teachers due to the incorporation of educational technology in the classroom. In this group, some of the most important items are knowledge, ability, attitude, and difficulty.

Cluster3: Represented in red, this group is related to the future themes and aspects that can promote the control of anxiety and stress in teachers due to the use of educational technology. Items include terms such as learning, experience, and instruction.

**Figure 5:**
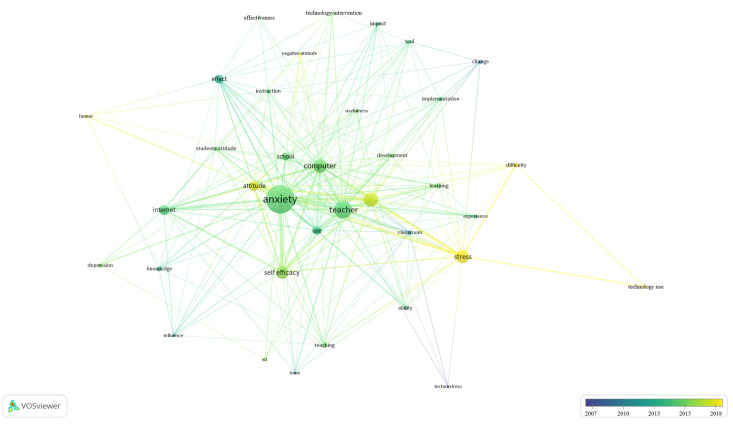
Bibliometric map of the time evolution of the 36 KW+ analyzed.

In addition, we also provide in Figure 5 a bibliometric map showing the time evolution related to teachers’ stress and anxiety associated with the use of educational technology, considering the 36 KW+ analyzed above. 

It can be observed that the colors tending to blue show little current interest in the scientific community, while those tending to yellow are currently generating greater relevance in terms of investigations in this field.

## 4. Discussion

This study presents the results of studies related to teacher stress and anxiety associated with the use of educational technology published between 2005 and 2019. Throughout the study, we were able to know the impact and the presence of scientific production within this field in the following databases: PubMed, WoS, and Scopus. The choice of these repositories was due to their importance in publications of scientific significance. 

Examining the results, research in this field has currently generated greater interest. This indicates that greater interest has been shown in this subject since 2015, an aspect that agrees with other studies [28]. However, the topic is still in an initial and expansion phase, as its expansion is not very significant with respect to other subjects. 

Scientific production in relation to the impact of educational technology on teachers’ mental health has developed and has been of great interest worldwide, although it has been found that its presence is not homogeneous among countries, being more numerous among Asian countries [28]. This situation suggests that future research should analyze whether the country influences the level of teacher stress due to the development of technology.

The studies analyzed show a mostly quantitative approach to evidence that educational technology encourages the development of stress and anxiety in teachers. Instead, there has been a lack of studies that analyze the different strategies to control these feelings among teachers, feelings that can have an impact on the exhaustion of their students [29]. 

The different articles selected focused mainly on the consequences on mental health due to the incorporation of educational technology in teaching practice. Among the main findings we can highlight the increase in anxiety and stress levels, as well as symptoms of exhaustion or depression in teachers, results that are consistent with numerous other studies [30].

The selected studies investigated the negative effects of the use of educational technology on teachers’ mental health. Considering the different studies, the results indicate mainly the symptoms of exhaustion, increased levels of anxiety, and perceived stress in teachers when using these tools. To a lesser extent, there were teachers who reported frustration in having to use educational technology in their teaching practice. 

The consequences of the use of these tools may be due to different factors, mainly the lack of training and education of teachers in educational technology, as well as the high levels of pressure to apply the technologies in daily teaching practice. This has repercussions on the reduction or improper application of these tools in the classroom [31]. 

The analysis of bibliometric maps has made it possible for us to identify the main research topics in this field, as well as the temporal evolution of the study of teachers’ stress and anxiety associated with the use of educational technology.

## 5. Conclusions

Over the past few decades, educational technology has become very important in education as well as in the professional growth of teachers. Moreover, the technologies have brought with them mental consequences derived from different factors, such as lack of training or pressure to use them [1].

This study aimed to provide an overview of scientific productivity in this field through the implementation of different bibliometric indicator strategies, enabling us to summarize and identify the main trends and emerging areas of research in this field. We must say that bibliometric indicators enable a quantitative and qualitative analysis of scientific production, namely, its impact [32]. The use of these indicators makes it possible to characterize very precisely the state of advancement of research and therefore to support decision-making on scientific policy. Different limitations were found during the review due to the scarce literature that exists in this field. Thus, the study should be extended to other databases—such as EMBASE, Medline, and PsycINFO—that provide a worldwide view of current research in this field. Likewise, there are other methodological limitations of the present study. The first is that the independent variable is not constant: using ICT now requires less (and different) technical knowledge and skill than it did 15 years ago. The second limitation is that, as the use of ICT has become more ubiquitous and familiar over time, it is reasonable to assume that the nature of stress and anxiety may be changing. The third limitation is that ownership of and familiarity with ICT devices varies across geography as well as across time. For example, the difference between Finland and Nigeria is considerable in this regard. The fourth limitation is that ICT is employed in very different ways for different ages and stages: its use in primary/elementary schools is very different from its use in universities—where it can also vary considerably across subject disciplines. The fifth limitation is that stress and anxiety caused by teachers’ perceptions of lack of training may not be the same as that caused by under-resourcing or unreliable kit or by the performance expectations of teachers’ managers. Such limitations are acknowledged as a methodological weakness of the study.

During the search for studies carried out, it was observed that the problem of teacher stress and anxiety related to educational technology has grown exponentially over time. In addition, differences have been detected between the most recent studies and those developed a few decades ago. Thus, concerns from decades ago have been reformulated thanks to subsequent studies in the field. We also see how new questions and factors have been emerging that threaten the integrity with which teachers implement technology and the quality of teaching and learning processes. This is the path to be followed to gradually reduce the levels of stress, anxiety, and frustration of teachers when using educational technology. Training is essential to achieve a professionalization of teachers with sufficient capacity to combat the challenges of instructional processes.

This topic is of great interest to teachers due to the current pandemic. In this sense, among future research lines, a comparative study could be carried out on how the use of technology has affected people’s mental health during the pandemic. Likewise, one of the challenges for teachers is to transmit to students the necessary tools so that their emotions do not affect their academic performance. This requires a high level of “perfectionist” personality and self-demand in the work carried out by teachers to improve their teaching practice.

Perfectionism can cause unnecessary stress on people. High levels of performance in the management of technologies can have a negative impact on the mental health of teachers, developing problems such as stress and/or anxiety. In the field of education, perfectionism is a growing and worrying phenomenon for experts and authorities. In fact, research from the University of Bath conducted with more than 40,000 students from universities in the United Kingdom, the United States, and Canada showed that it has increased by 33% since 1989 [33]. Faced with this phenomenon, teachers must transmit to their students the necessary tools to learn to assimilate failure and thus become resilient young people who focus more on ways to overcome their problems than on these inconveniences.

## Figures and Tables

**Table 1:** Analysis of selected studies.

Study	Year	Methodology	Main Contributions	Country
Pillay, H., Goddard, R. & Wilss, L. [12]	2005	Quantitative	The use of technology resources in the classroom affects negatively on teachers’ wear and tear, stress, and burnout.	Australia
Al-Fudail, M. & Mellar, H. [13]	2008	Qualitative	Problems experienced by teachers with the use of technology include increased levels of stress.	United Kingdom
La Paglia, F., Caci, B. & La Barbera, D. [14]	2008	Quantitative	Low technology training produces anxiety in teachers.	Italy
Agbatogun, A.O. [15]	2010	Quantitative	Anxiety influences the teacher’s attitude to use technology.	United Kingdom
Bolandifar, S. & Noordin, N. [16]	2015	Quantitative	Teacher anxiety should be eliminated by using computers and Internet in the classroom.	Malaysia
Jena, R.K. [17]	2015	Quantitative	Pressure to use technology has increased teacher stress.	India
Çoklar, A.N., Efíltí, E., Sahín, Y.L., &Akçay. A. [18]	2016	Quantitative	Insufficient technology training increases teachers’ stress levels.	Turkey
Joo, Y.J, Lim, K.Y. & Kim, N.H. [19]	2016	Quantitative	Technostress impacts negatively on intentions to use technology.	South Korea
Syvänen, A., Mäkiniemi, J.P., Syrjä, S., Heikkilä-Tammi, K., &Viteli, J. [20]	2016	Quantitative	Limited technology inclusion in the classroom is due to high levels of technostress.	Finland
Revilla Muñoz, O., Alpiste Peñalba, F., Fernández Sánchez, J. & Santos, O.C. [21]	2017	Quantitative	Anxiety is reduced when taking courses on problem solving skills with technology.	Spain
Tucker, C. [22]	2018	Quantitative	Teachers are exhausted due to all the innovation and use of new technology-based teaching strategies.	United States
Awofala, A.O.A., et al. [23]	2019	Mixed	Anxiety reduces experiences with technology and requires more attention.	Nigeria
Goebel, D.K. & Carlotto, M.S. [24]	2019	Quantitative	One of the variables affecting teacher stress is the need for updating and training in technology.	Brazil
Hassan, N., et al. [25]	2019	Quantitative	Technology insecurity impacts on teachers’ stress patterns.	Malaysia
Othman, Z. & Sivasubramaniam, V. [26]	2019	Quantitative	Levels of anxiety, stress, or depression are increasing due to using technology.	Malaysia
Wang, X. & Li, B. [27]	2019	Quantitative	Factors such as lack of technology knowledge may increase stress.	China

## Data Availability

No new data were created or analyzed in this study. Data sharing is not applicable to this article.
